# IgG Anti-high-Density Lipoproteins Antibodies Discriminate Between Arterial and Venous Events in Thrombotic Antiphospholipid Syndrome Patients

**DOI:** 10.3389/fmed.2019.00211

**Published:** 2019-09-26

**Authors:** Savino Sciascia, Irene Cecchi, Massimo Radin, Elena Rubini, Ana Suárez, Dario Roccatello, Javier Rodríguez-Carrio

**Affiliations:** ^1^Center of Research of Immuno-pathology and Rare Diseases, Coordinating Center of Piemonte and Valle d’Aosta Network for Rare Diseases, and SCDU Nephrology and Dialysis, Department of Clinical and Biological Sciences, S. Giovanni Bosco Hospital, University of Turin, Turin, Italy; ^2^Department of Functional Biology, Immunology Area, Faculty of Medicine, University of Oviedo, Oviedo, Spain; ^3^Instituto de Investigación Sanitaria del Principado de Asturias (ISPA), Oviedo, Spain; ^4^Bone and Mineral Research Unit, Instituto Reina Sofía de Investigación Nefrológica, REDinREN del ISCIII, Hospital Universitario Central de Asturias, Oviedo, Spain

**Keywords:** antiphospholipid syndrome, thrombosis, anti-HDL, autoimmunity, autoantibodies

## Abstract

**Introduction:** Recurrent thrombotic events are a hallmark of Antiphospholipid Syndrome (APS). However, biomarkers to identify if a patient with antiphospholipid antibodies (aPL) is at higher risk to develop an arterial or a venous event are lacking. Recently, the pathogenic role of anti-high-density lipoproteins antibodies (anti-HDL) in the occurrence of cardiovascular disease (CVD) in autoimmunity has emerged. The aim of the present study was to evaluate the presence of IgG anti-HDL antibodies in a cohort of thrombotic APS patients and to investigate their association with clinical outcomes.

**Methods:** Serum levels of IgG anti-HDL antibodies, total IgG, and complete aPL profile were assessed in 60 APS patients and 80 healthy donors (HDs) by immunoassays.

**Results:** Higher levels of IgG anti-HDL were found in APS patients compared to HDs (*p* < 0.001), even after correcting for total IgG levels (*p* < 0.001). No associations with treatments or traditional cardiovascular risk factors, except for smoking habit (*p* < 0.0001), were found. Patients who experienced at least one arterial event (*n* = 30) had significantly higher levels of anti-HDL antibodies when compared to patients with venous thrombosis (*n* = 30, *p* = 0.046), this difference being stronger when adjusting for total IgG (*p* = 0.007). Additionally, patients tested positive for antiphosphatidylserine/prothrombin (IgG/IgM) antibodies had significantly higher levels of anti-HDL antibodies (*p* = 0.045).

**Conclusions:** Increased levels of IgG anti-HDL antibodies can be found in APS, mainly in patients with arterial thrombosis, independently of aPL antibodies and traditional risk factors. These findings point to a role of anti-HDL antibodies in APS and support their use as a potential biomarker for arterial thrombotic events.

## Introduction

Antiphospholipid Syndrome (APS) is the most common acquired thrombophilia. At the clinical level, APS is defined by the occurrence of thrombotic events, with the peculiar trait of potentially involving both arteries and veins and/or pregnancy morbidity, in individuals found to be persistently positive for antiphospholipid antibodies (aPL), including: lupus anticoagulant (LA), anti-cardiolipin (aCL), and anti-β2glycoprotein I (anti-β2GPI) antibodies (1). Additionally, premature cardiovascular disease (CVD) and atherosclerotic development have been proven to be more prevalent in APS compared to the general population (2, 3).The mechanisms underlying thrombosis and CVD in APS patients are not completely understood, but recent evidences have brought to light the existence of a complex interplay between conventional cardiovascular risk factors and disease-specific features, such as the presence of autoantibodies (4).

Interestingly, several studies have reported that aPL might be able to cross-react with lipoproteins and their components, contributing to endothelial dysfunction, enhancing atherosclerosis, and ultimately leading to CVD progression (5, 6). However, the clinical relevance of such findings is unknown. In addition, recent studies have discovered the existence of a heterogeneous group of autoantibodies specifically directed against lipoproteins and their components, namely IgG anti-high-density lipoproteins antibodies (anti-HDL), which have been demonstrated to impair the anti-inflammatory and anti-oxidative roles exerted by HDL-cholesterol (7). Higher anti-HDL levels have been described in a broad range of autoimmune diseases (8–10). However, whether anti-HDL antibodies may be associated with clinical features in APS remains unclear. Critically, while it is known that the presence of aPL confers a high risk for thrombosis (11), biomarkers to if a patient is at higher risk to develop an arterial or a venous event are lacking. The aim of the present study was to evaluate the presence of IgG anti-HDL antibodies in a cohort of thrombotic APS patients and to investigate if these antibodies can discriminate between arterial and venous thrombosis.

## Methods

### Ethics Statement

The study protocol, involving human samples, was performed in compliance with the Declaration of Helsinki and reviewed and approved by the Institutional Review Boards (IRBs) from the University of Turin and the University of Oviedo. All participants gave written informed consent prior enrolment.

### Patients

This cross-sectional study included 60 APS patients attending the Giovanni Bosco Hospital, Turin, Italy. Inclusion criteria comprehended: patients with persistent aPL positivity and that fulfilled the Sydney criteria for thrombotic APS (venous and/or arterial) (1). A group of 80 age- and sex-matched healthy individuals from the same population was recruited as healthy donors (HDs). Medical records from APS were retrospectively revised in order to register clinical characteristics, including previous episodes of venous and/or arterial thrombosis. Blood samples were collected after the first thrombotic event.

### Antiphosholipid Antibodies Testing

The aPL profile included LA, aCL, and anti-ß2GPI, and anti-phosphatidylserine/prothrombin (aPS/PT) antibodies. The aCL, anti-ß2GPI, aPS/PT (IgG and IgM) were semi-quantitatively assayed using a commercial ELISA kit by Inova Diagnostics, Inc (San Diego, CA, United States).

Plasma samples were tested for the presence of LA according to the recommended criteria from the International Society on Thrombosis and Haemostasis Subcommittee on Lupus Anticoagulant/Phospholipid-Dependent Antibodies (12, 13).

### IgG Anti-HDL Antibodies

IgG antibodies against HDL were measured in all serum samples by ELISA as previously described (9). ELISA plates (Maxisorp, Nunc) were coated overnight (4°C) with 20 μg/ml human HDL-cholesterol (Sigma) in 70% ethanol (test half) or ethanol alone (control half). Then, plates were blocked with PBS + 1% BSA (Sigma) for 1 h at room temperature and washed with PBS. Serum samples (1:50-diluted in PBS + 0.1% BSA), and standard curves from pooled sera (diluted 1:16 to 1:512) were incubated in both plate halves for 2 h at room temperature. Plates were then washed twice with TBS and alkaline phosphatase-conjugated anti-human IgG (1:1,000) (Immunostep) was added for 1 h. Finally, p-nitrophenylphosphate (Sigma) in diethanolamine buffer (pH 9.8) was added and absorbance at 405 nm was recorded. Anti-HDL Arbitrary Units (AU) were calculated for each sample according to the standard curves. Intra- and inter-assay reproducibility for our assay was 10 and 13%, respectively. Total IgG was quantified by conventional ELISA techniques and AU values obtained from the anti-HDL ELISA were corrected using total IgG levels (anti-HDL/IgG). The positivity to anti-HDL antibodies was evaluated using the 90th percentile of the anti-HDL/IgG in HDs (= 12.94) as cut-off (9). This cut-off provided the following analytical estimates in the present study: sensitivity = 0.46, specificity = 0.90, and positive predictive value = 0.91.

### Statistical Analysis

Categorical variables are presented as number (%) and continuous variables are presented as mean (S.D.). Differences were evaluated by the chi-squared test, Fisher's exact test or the unpaired *t*-tests (Mann–Witney or Kruskal–Wallis), as appropriate. Spearman rank's test was used to analyze correlations. ROC curves were performed to evaluate the association between anti-HDL positivity and thrombotic outcomes, and the corresponding area under the curve (AUC) and its 95% confidence intervals and *p*-values were computed. A two-sided *P* < 0.050 was considered as statistically significant. All statistical analyses were performed using SPSS version 19.0 (IBM, Armonk, NY, USA).

## Results

### IgG Anti-HDL Antibodies in APS Patients

Sixty APS patients were enrolled in this study, 43 (71.6%) patients being primary APS patients (PAPS) and 17 (28.4%) patients having a concomitant diagnosis of SLE, according to the recently approved classification criteria of the European League Against Rheumatism and the American College of Rheumatology, which includes at least one positive antinuclear antibody test and the combination of a number of clinical and immunological criteria (14). All 60 patients had at least one thrombotic event: 30 (50%) previous arterial events and 37 (61.6%) venous events (seven patients experienced recurrent events, both venous and arterial). For the purpose of this study the analysis was performed taking into account the first thrombotic event, meaning that 30 patients were considered as arterial thrombotic APS patients and 30 patients as venous thrombotic APS patients. No differences have been found between PAPS and secondary APS patients (SAPS) in terms of age (*p* = 0.190), gender (*p* = 0.21), cardiovascular risk factors [including arterial hypertension (*p* = 0.281), hyperlipidemia (*p* = 0.670), and smoking habit (*p* = 0.290)], C-reactive protein values (*p* = 0.540) and HDL cholesterol levels (*p* = 0.721), and aPL profile (all *p* > 0.050). In addition, no differences have been found when looking at the levels of anti-HDL antibodies between these two groups (*p* = 0.570), even when correcting for total IgG levels (*p* = 0.860). One SAPS patient had undergone B-cell depletion therapy within 1 year prior to blood sample collection. Full demographics and clinical characteristics of the study cohort are detailed in Table 1.

**Table 1:** Demographic, clinical, and laboratory characteristics of the APS patients and HDs included in the study.

**Patients characteristics**	**HDs (*n* = 80)**	**APS patients (*n* = 60)**
Female sex (*n*, %)	60 (75)	43 (71.6)
Age (mean, S.D.), years	48.6 ± 10.4	50 ± 10.8
Primary APS patients (*n*, %)	0 (0)	43 (71.6)
Concomitant diagnosis of SLE (*n*, %)	–	17 (28.3)
Disease duration since APS diagnosis (mean, S.D.), years	–	11.7 ± 7.5
Thrombosis (*n*, %)	0 (0)	60 (100)
Venous thrombosis (*n*, %)	–	37 (61.6)^*^
Arterial thrombosis (*n*, %)	–	30 (50)
Pregnancy morbidity (*n*, %)	0 (0)	1 (1.6)
Arterial hypertension (*n*, %)	4 (5)	22 (36.6)
Hyperlipidemia (*n*, %)	0 (0)	19 (31.6)
Smoking (*n*, %)	9 (11.2)	5 (8.3)
Obesity (BMI > 30) (*n*, %)	0 (0)	8 (13.3)
LA (*n*, %)	0 (0)	49 (81.6)
aCL IgG/M (*n*, %)	0 (0)	38 (63.3)
Anti-β2 GPI IgG/IgM (*n*, %)	0 (0)	29 (43)
aPS/PT IgG/IgM (*n*, %)	0 (0)	29 (48.3)
Triple aPL positivity (*n*, %)	–	20 (33.3)
Total cholesterol levels (mean, S.D.), mg/dl	145 ± 32.2	187.4 ± 51
HDL-cholesterol levels (mean, S.D.), mg/dl	72 ± 15.3	64 ± 9.1
Triglycerides levels (mean, S.D.), mg/dl	104 ± 26.2	98.7 ± 28.6
CRP levels (mean, S.D.), mg/dl	0.23 ± 0.2	0.45 ± 0.2
Statins (*n*, %)	0 (0)	20 (33.3)
Anti-hypertensive drugs (*n*, %)	3 (5)	19 (31.6)
HCQ (*n*, %)	0 (0)	15 (25)
Anticoagulant agents (*n*, %)	0 (0)	33 (55)
Antiaggregant agents (*n*, %)	0 (0)	36 (60)
B-cell depletion agent (Rituximab) (*n*, %)	0 (0)	1 (1.2)

* *Seven patients experienced recurrent thrombotic events, both venous and arterial. For the purpose of this study the analysis was performed taking into account the first thrombotic event, meaning that 30 patients were considered as arterial thrombotic APS patients and 30 patients as venous thrombotic APS patients. APS, antiphospholipid syndrome; HDs, healthy donors; SLE, systemic lupus erythematosus; BMI, adult body mass index; LA, lupus anticoagulant; aCL, anti-cardiolipin antibodies; anti-β2 GP1, anti-β2glycoprotein I antibodies; aPS/PT, antiphosphatidylserine/prothrombin antibodies; aPL, anti-phospholipid antibodies; CRP, C-reactive protein; HCQ, hydroxychloroquine*.

**Figure 1:**
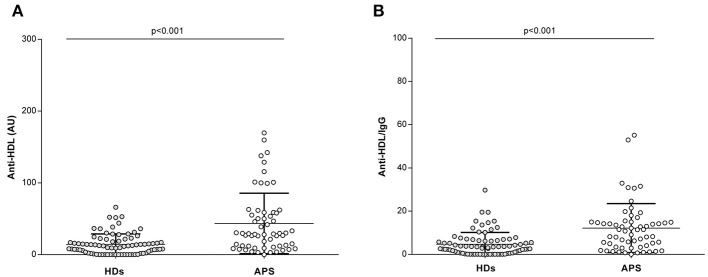
Anti-HDL antibodies in APS patients. Levels of IgG anti-HDL antibodies measured as AU **(A)** or normalized after total IgG correction **(B)** in APS patients (*n* = 60) and HDs (*n* = 80). Bars indicate 25th, median and 75th percentiles. Differences were assessed by Mann–Whitney *U*-tests.

Higher levels of IgG anti-HDL were found in APS patients compared to HDs [mean 46.1 (SD ±69.8) vs. mean 14.3 (SD ±14.5), respectively; *p* < 0.001] (Figure 1A). Anti-HDL levels were found to be increased even after correcting for total IgG levels [mean 12.6 (SD ±16.3) vs. mean 4.7 (SD ±5.5), respectively; *p* < 0.001] (Figure 1B).

The levels of anti-HDL antibodies were not associated with traditional CV risk factors, including arterial hypertension (*p* = 0.800), hyperlipidaemia (*p* = 0.102), diabetes (*p* = 0.700), and high body mass index (*p* = 0.800). Anti-HDL levels were not correlated to HDL levels in APS (*r* = 0.090, *p* = 0.630) nor in HDs (*r* = 0.110, *p* = 0.370). Similar results were retrieved when APS patients were analyzed separately as PAPS and SAPS (*r* = 0.120, *p* = 0.561; and *r* = −0.050, *p* = 0.710; respectively). On the contrary, higher levels of anti-HDL antibodies were observed in smokers compared with non-smokers [mean 112.42 (SD ±202.2) vs. mean 36.6 (SD ±38.7); *p* < 0.0001], even after adjusting for total IgG levels [anti-HDL/IgG: mean 16.2 (SD ±26.2) vs. mean 10.9 (SD ±11.1); *p* = 0.012]. Finally, levels of total IgG were found to be similar between APS patients and HDs [mean 382.23 (SD ±154.88) vs. mean 333.63 (SD ±115.37); *p* = 0.262] and not related to clinical parameters, thrombosis status or treatments (all *p* > 0.050).

Overall, an increased prevalence of IgG anti-HDL antibodies can be observed in APS patients. Traditional CV risk factors were not related to anti-HDL levels, with the exception of smoking habit.

### IgG Anti-HDL Antibodies and Clinical Features in APS Patients

When separating for individual aPL positivities, patients tested positive for aPS/PT (IgG/IgM) antibodies had significantly higher levels of anti-HDL [mean 53.1 (SD ±81.1) vs. mean 20.7 (SD ±17.6); *p* = 0.045], which did not reach statistical significance after adjusting for total IgG (*p* = 0.151). No differences were observed with the rest of aPL tested. Similarly, no differences were observed when the different isotypes of aPL antibodies (IgG/IgM) were entered separately in the analyses (all *p* > 0.050). Additionally, no differences were observed between patient with primary APS and those with a concomitant autoimmune diagnose. Finally, IgG anti-HDL levels were not associated with treatments received (all *p* > 0.050).

ROC analyses (Supplementary Figure 1) revealed a good discriminative power of the anti-HDL positivity to the presence of thrombosis (both arterial and venous) (AUC ROC [95% CI], *p*: 0.751 [0.633, 0.870], *p* < 0.001), hence strengthening their role as potential biomarker.

**Figure 2:**
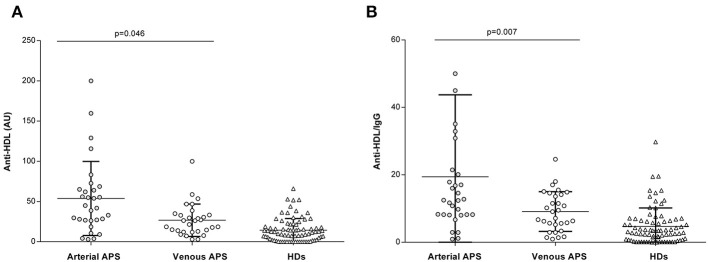
Anti-HDL antibodies and clinical features in APS. Levels of IgG anti-HDL antibodies measured as AU **(A)** or normalized after total IgG correction **(B)** in APS patients with arterial thrombosis (*n* = 30, gray dots), venous thrombosis (*n* = 30, open dots) (Table 1) and HDs (*n* = 80, open triangles). Differences were assessed by Kruskal–Wallis tests and *p*-values indicated were derived from Dunn–Bonferroni tests for multiple comparisons.

Finally, when separating patients for the different thrombotic manifestations of APS (arterial vs. venous), we observed that patients who experienced at least one arterial event had significantly higher levels of anti-HDL when compared to patients with venous thrombosis [mean 53.1 (SD ±94.1) vs. mean 34.3 (SD ±28.9), respectively; *p* = 0.046] (Figure 2A). This difference became stronger when adjusting for total IgG levels [anti-HDL/IgG: mean 13.1 (SD ±16.7) vs. mean 9.5 (SD ±6.6); *p* = 0.007] (Figure 2B, right panel). No significant difference was found in total IgG levels between patients who experienced an arterial or a venous thrombotic event [mean 378.2 (SD ±148) vs. mean 375.5 (SD ±136.3), respectively; *p* = 0.950]. Importantly, none of the aPL antibodies differ between arterial and venous thrombosis, and no differences were observed for clinical features and treatments received (all *p* > 0.050). Furthermore, the distribution of traditional CV risk factors was similar between both subsets of APS patients (all *p* > 0.050). Taken together, our results confirm that anti-HDL antibodies were associated with clinical outcomes in APS, as aPS/PT positivity and arterial thrombotic manifestation, independently of other clinical features, hence suggesting its potential use as biomarkers.

## Discussion

Autoimmune and rheumatic diseases are associated with a higher prevalence of CV morbidity and mortality, mainly due to an accelerated atherosclerotic process (15, 16). Far from the early conception of a natural, evolutive aging-related process, a compelling body of evidence supports that atherosclerosis and atherothombosis are dynamic and complex conditions resulting from an inextricable link of multiple pathogenic factors that trigger and perpetuate the vascular damage and impair its reparative mechanisms (17). Immune (systemic) mediators and autoantibodies emerge as crucial pathogenic players in this scenario. Therefore, atherosclerosis and atherothrombosis are now seen as the consequence of the interplay between traditional risk factors and autoimmune-related mechanisms (18). As a consequence, the presence of traditional CV risk factors alone cannot fully explain the increase CV morbidity (18). In addition, although the presence of chronic inflammation and the production of disease-specific antibodies, such as aPL, play a crucial role in the pathogenesis of endothelial dysfunction and thromboembolic manifestations, the exact mechanisms involved are largely unknown and recent evidence seem to point to the involvement of novel mediators. The results presented in this paper suggest the involvement of a new player, anti-HDL antibodies, in this scenario. If validated in larger prospective studies, our findings might also support the potential use anti-HDL antibodies as biomarkers for the early identification of patients with arterial thrombosis.

Despite previously being considered as mere bystanders, lipoproteins are important and active mediators of pathogenic processes such as inflammation, oxidative stress, and metabolic traits in autoimmunity. In fact, altered lipoprotein levels and/or functionality have been reported in several autoimmune conditions. In recent years, a growing body of evidence has highlighted the involvement of anti-HDL antibodies as a bridge between humoral immune-response, lipid dysfunction, oxidative status, and clinical outcomes in rheumatic disorders (8, 19, 20). To date, in the specific setting of APS, limited data are available regarding lipid profile and the association of anti-HDL with clinical features of the disease had been unexplored. Previous studies have demonstrated increased levels of anti-HDL in APS patients when compared to HDs and SLE patients without APS (6, 7, 21) In addition, increased levels of anti-HDL were found to be inversely correlated with the levels of paraoxonase-1 (PON1), accounting for the antioxidant effect of HDL (22). In line with the data available in the literature so far, our study showed that APS patients presented significantly increased levels of IgG anti-HDL when compared to HDs, hence confirming this result in the larger APS cohort analyzed until date. Interestingly, our results went further by confirming that this result remained after correcting for total IgG levels, thus suggesting that higher levels of anti-HDL antibodies cannot be attributed to a general over-activation of the immune system in the context of autoimmunity, but to the specific production of these antibodies. Additionally, the emergence of anti-HDL antibodies in APS was not linked to the positivity of other aPL antibodies or treatments. Moreover, anti-HDL were not related to traditional CV risk factors in APS patients, in line with previous findings in SLE and RA cohorts by our group and others (9, 21), thus strengthening their role as independent, complimentary biomarkers.

A remarkable finding from our study was the association with arterial thrombosis. Even if deep vein thrombosis represents the most common feature of APS, arterial events constitute the most dangerous and potentially life-threatening manifestations of the disease, affecting primarily the central nervous system and young adults < 50 years old (3, 23, 24) Although some progresses have been made in order to identify those patients who are at higher risk for developing arterial events, it still represents an urgent unmet clinical need (25–27).

When analyzing the association between anti-HDL and clinical manifestation of APS in our study, we found statistically significant higher levels of IgG anti-HDL in those patients who have a history of arterial events. This result suggests that arterial APS patients might display a prominent impairment of the anti-atherogenic function of HDL, which represent a key step in endothelial dysfunction, oxidative stress, atherosclerotic plaque formation and progression, ultimately leading to atherothrombotic manifestations. Importantly, no differences in other clinical and laboratory parameters have been found between these groups of patients. If our observation were confirmed in larger prospective studies, IgG anti-HDL might represent an additional tool to CV risk factors profiling in the identification and management of “high-risk patients,” which might guide the therapeutic strategies. In fact, the recent RAPs and TRAPs trials (28, 29) have reported different results when it comes to using new agents for venous and arterial events, hence strengthening the validity of our findings as biomarkers for the clinical setting. In this context, “high risk patients” might benefit of combined thrombo-prophylactic therapy as primary (e.g., anti-platelets and hydroxycholoroquine) or secondary prophylaxis (anti-platelets/hydroxycholoroquine associated to VKA) (30); similarly, they might be discouraged to the use of direct oral anticoagulants (31, 32). However, the real impact of IgG anti-HDL testing on therapeutic patients' management is not addressable in this study due to its cross-sectional design.

As mentioned above, our data showed that higher levels of anti-HDL were found in smokers. The link between smoke and autoimmunity has been already described in a wide range of pathologic conditions (33). Cigarette smoking exerts several pro-inflammatory effects, increasing oxidative stress, inducing the release of intracellular antigens, the augmentation of auto-reactive B-cells activity and an overall production of autoantibodies, including aPL (34). In this context, smoking might represent an important environmental trigger for anti-HDL production, and a potential mechanistic link between this risk factor and the occurrence of either thrombotic-embolism or atherosclerosis development, the main clinical outcomes related to anti-HDL in the literature. However, further analyses are needed in order to clarify this possible association from a mechanistic point of view and its clinical impact in APS.

Finally, our study shed new light into the associations between anti-HDL and disease-related autoantibodies. On the one hand, early studies from other groups reported certain degree of cross-reactivity between aCL and anti-HDL antibodies (6), although this was not confirmed in other studies (6). Importantly, previous analyses on these antibodies in APS were performed in low sample size populations. Our findings revealed no association between anti-HDL and aCL antibodies, challenging the previous notion. This is in line with previous studies from our group when analyzing other disease-related autoantibodies positivity (8–10). Recent advances have brought to light the existence of a heterogeneous group of pathogenic autoantibodies in APS. Among them, aPS/PT antibodies have been proven to have a clinical independent relevance in this setting (35), confirmed in international studies by our group and others (36, 37). In our analysis, despite anti-HDL levels not showing any association other aPL antibodies, patients tested positive for aPS/PT antibodies exhibited higher levels of anti-HDL. As the clinical role of aPS/PT is a rising topic, particularly when other aPL tests are negative, this association could be of special interest and could make a case for anti-HDL as new potential biomarkers in this specific subset of patients.

This study has potential limitations, including mainly the cross-sectional retrospective design and the limited sample size. Indeed, prospective larger studies are needed to confirm these findings. However, our study documented, in line with the data available in the literature about the presence of IgG anti-HDL antibodies in APS and expanded the current knowledge about the emerging role of these autoantibodies in autoimmune-mediated diseases and CVD. Moreover, whether anti-HDL antibodies could be also associated with other surrogate markers of CVD (such as subclinical atherosclerosis), in addition to their association with thrombosis, remains to be elucidated. Similarly, patients exhibiting both arterial and venous events were not analyzed separately due to sample size concerns. Further studies may elucidate if these patients show a different/intermediate profile of anti-HDL antibodies. On the other hand, it may be interested to analyze whether anti-HDL antibodies may be linked to aPL-pregnancy related complications. However, due to sample size concerns and potential clinical differences (38), this was not explored in our study. Additionally, although differences in hyperlipidemia were observed between patients and controls, the lack of association between lipid profiles and anti-HDL levels, and the relatively small differences in lipid levels between these two groups lead us to think that this do not represent a major limitation for our findings. This point is also supported by the existing literature findings (8, 39–44). In summary, to the best of our knowledge, this is the first study informing an association between anti-HDL antibodies and thrombotic outcomes in APS patients. Our study warrants future pathogenic studies are needed to confirm such observations. Moreover, our findings support that anti-HDL might represent a promising tool for risk management and assessment and a reliable biomarker for the early identification of arterial thrombotic events. Despite some preliminary evidence (6, 45), exploring the role of autoantibodies against lipoprotein components is still intriguing in the APS setting, as their increased thrombotic and atherothrombotic profile might support the need of targeted specific approaches.

## Data Availability Statement

The raw data supporting the conclusions of this manuscript will be made available by the authors, without undue reservation, to any qualified researcher.

## Author’s Note

An interim version of this study has been presented as a poster at the 2018 American College of Rheumatology (ACR) annual meeting (Abstract Number 168). The authors have expanded the sample and analyses performed upon.

## Author Contributions

SS, IC, MR, and ER were in charge of patients' recruitment, clinical data collection, and analysis of the results. AS and DR contributed to the study conception, analysis, and discussion of the results. SS and JR-C conceived the study and designed the study protocol. SS, IC, and JR-C drafted the manuscript. JR-C performed the experimental procedures and edited the manuscript. All authors read and approved the final version of the manuscript.

### Conflict of Interest

The authors declare that the research was conducted in the absence of any commercial or financial relationships that could be construed as a potential conflict of interest.
