# Supplementary material for: IgG Anti-high-Density Lipoproteins Antibodies Discriminate Between Arterial and Venous Events in Thrombotic Antiphospholipid Syndrome Patients
Source: Front Med (Lausanne). 2019 Sep 26;6:211. doi: 10.3389/fmed.2019.00211 (PMC6775216; doi:10.3389/fmed.2019.00211)

## ONLINE SUPPLEMENTARY MATERIAL

**Supplementary Figure 1: ROC analysis.** The discriminative power of anti-HDL positivity to identify the presence of thrombosis was evaluated by means of a ROC curve (red line). AUC ROC [95% CI], p: 0.751 [0.633, 0.870], p<0.001.

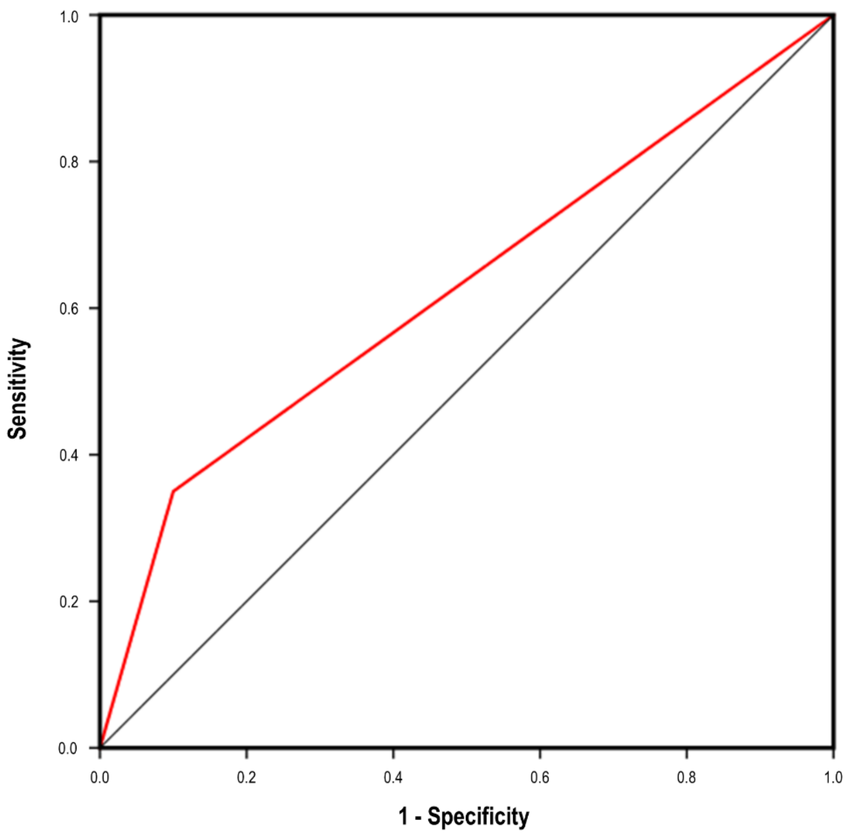

Supplement: Supplementary file 1 [file Image_1.pdf]
